# Masked emotions: Do face mask patterns and colors affect the recognition of emotions?

**DOI:** 10.1186/s41235-022-00380-y

**Published:** 2022-04-08

**Authors:** Olesya Blazhenkova, Kivilcim Dogerlioglu-Demir, Robert W. Booth

**Affiliations:** 1 Faculty of Arts and Social Sciences, Sabanci University, Orta Mahalle, Üniversite Caddesi No:27, 34956 Tuzla-Istanbul, Turkey; 2 Sabanci Business School, Sabanci University, Orta Mahalle, Üniversite Caddesi No:27, 34956 Tuzla-Istanbul, Turkey

**Keywords:** COVID-19, Face mask, Angularity versus curvature, Black versus white, Face mask perceptions

## Abstract

Previous research has shown that face masks impair the ability to perceive social information and the readability of emotions. These studies mostly explored the effect of standard medical, often white, masks on emotion recognition. However, in reality, many individuals prefer masks with different styles. We investigated whether the appearance of the mask (pattern: angular vs. curvy and color: black vs. white) affected the recognition of emotional states. Participants were asked to identify the emotions on faces covered by masks with different designs. The presence of masks resulted in decreasing accuracy and confidence and increasing reaction times, indicating that masks impair emotion recognition. There were no significant effects of angularity versus curvature or color on emotion recognition, which suggests that mask design may not impair the recognition beyond the effect of mere mask wearing. Besides, we found relationships between individual difference variables such as mask wearing attitudes, mask design preferences, individual traits and emotion recognition. The majority of participants demonstrated positive attitudes toward mask wearing and preferred non-patterned black and white masks. Preferences for white masks were associated with better emotion recognition of masked faces. In contrast, those with negative attitudes toward masks showed marginally poorer performance in emotion recognition for masked faces, and preferred patterned more than plain masks, perhaps viewing masks as a fashion item rather than a necessity. Moreover, preferences to wear patterned masks were negatively related to actual wearing of masks indoors and perceived risks of COVID.

## Significance statement

In response to the COVID-19 pandemic, many people around the world wear standard, often white, medical face masks in public, covering a major part of the face. However, as the pandemic continued, and mask wearing became the ‘new normal,’ individuals opted for stylish face masks; thus, many brands produced non-medical face masks with vibrant patterns and motifs. As face masks have become a staple in our wardrobes, more and more people use masks with a variety of designs. While previous research mostly studied the effects of standard white masks, we investigated whether the appearance of the mask (the pattern-angular vs. curvy and the color-black vs. white) affects the recognition of emotional states. Our findings suggest that these designs may not further impair readability of emotions, at least not much more than standard masks. We also found that negative attitudes toward mask wearing were negatively related to accuracy and confidence in emotion recognition of only masked but not unmasked faces. This finding is important as it suggests that those who have unfavorable mask perceptions might have reduced sensitivity to reading emotions specifically on masked faces. Our results further demonstrated that most individuals preferred plain black or white masks. Interestingly, those with negative attitudes toward masks and those who estimated COVID-19 risks to be lower preferred patterned masks, perhaps viewing masks as an accessory rather than a necessity. The current study opens avenues for future studies on the effects of non-standard masks on emotion recognition.

## Introduction

In early 2020, COVID-19 turned into a global public health emergency, affecting the lives of millions of people. Wearing a face mask in public became a requirement in many countries (Silchenko & Visconti, 2021) as one of the major means to prevent the transmission of the virus (Marini et al., 2021). The World Health Organization suggests wearing a mask for both preventing spread of the COVID-19 and protection purposes especially in low air circulation places such as schools, closed buildings, etc. (WHO, 2020). More than a year after the COVID-19 pandemic emerged, face mask use continues. Some even refer to the usage of face masks as the ‘new normal’ (Corpuz, 2021). Amid widespread recommendation to wear face masks in public, there is resistance from some people to do so. Despite the clear benefits of the practice, such a resistance stems from reasons such as inconvenience of wearing masks, perception of an infringement of independence or perceived undesirable appearance (Howard, 2020). As a result, many people opted for individualized self-made or branded stylish face masks that may have shifted the perception of masks as accessories rather than medical devices. In fact, many brands have produced non-medical face masks with vibrant patterns and motifs. As face masks make social interactions more difficult, the social barriers they create were also reported to be one of the main determinants that shape negative face mask perception (Howard, 2020; Taylor & Asmundson, 2021). Many studies showed that face masks inhibit the capability to perceive a lot of the social information expressed by faces (Bani et al., 2021; Carbon, 2020; Freud et al., 2020; Marini et al., 2021).

Faces provide crucial information that allows for identifying others; it is the most distinguishing and commonly used body part to read a person’s identity, age, sex, trait characteristics, intentions and emotions (Bruce & Young, 1986; Willis & Todorov, 2006). Individuals mostly rely on the whole face when recognizing an emotion (Baron-Cohen et al., 1997a; Smith et al., 2005; Wegrzyn et al., 2017) and whether the focus is on the upper or lower facial areas depends on the specific emotion (Ekman, 1972). Therefore, it is not surprising that occlusion of salient face regions may significantly impair readability of certain emotions (Bassili, 1979; Roberson et al., 2012).

Past research has demonstrated that the eyes are the most salient part of the face. Research in face processing showed that individuals pay the utmost attention to eyes when processing a face (Argyle, 1970; Blazhenkova, 2017; Goldstein & Mackenberg, 1966; Itier et al., 2007; James et al., 2010; Janik et al., 1978). In effect, studies that employ facial stimuli that use both positive and negative contrast demonstrate that the eye region is the major factor in facial recognizability (Gilad et al., 2009). Although the contrast of the rest of the face can affect face processing, just putting the eyes in 'positive' restores the recognizability of the face (Fisher et al., 2016). Baron-Cohen et al. (1997a) created an emotion recognition test (‘Reading the Mind in the Eyes Test’) based on the eyes area cropped out of the face that aimed to test ‘theory of mind’ or social sensitivity. This study examined the role of eyes in attribution of mental states (happy, sad, angry, etc.) and complex mental states (admiration, interest, thoughtfulness, etc.) and found that for the basic emotions, the whole face is more informative than either the eyes or the mouth. However, for the complex mental states, eyes were as communicative as the whole face.

The mouth region was also found to be a salient part of the face involved in expressing emotions (Beaudry et al., 2014; Blais et al., 2017; Blazhenkova, 2017; Bombari et al., 2013; Calvo et al., 2014; Eisenbarth & Alpers, 2011; Smith et al., 2005), albeit secondary to the eye region (Blazhenkova, 2017; McKelvie, 1995; Pellicano et al., 2006). Covering the lower portion of the face was found to impede the perception of happiness since smiling—a sign of happiness—is characterized by a contraction of the zygomaticus major muscle that extends from the cheekbone to the corners of the mouth (Fischer et al., 2012). When individuals were asked to focus on the upper region of a happy face (vs. the lower region of the face), detection of happiness was severely impaired as the eyes are not as relevant as the mouth for the identification of happiness (Bombari et al., 2013). Further, some eye-tracking studies reveal that the mouth area is more fixated during the recognition of happiness (Beaudry et al., 2014; Eisenbarth & Alpers, 2011). Though most studies show that the mouth area is focused when happiness is detected (Blais et al., 2012; Eisenbarth & Alpers, 2011; Fischer et al., 2012; Kotsia et al., 2008), when ‘Duchenne smiles’ are observed (Okazaki et al., 2021; Sheldon et al., 2021), the eyes alone are diagnostic, as the term *Duchenne* refers to smiles in which both the muscle that orbits the eye and zygomaticus major are active.

Taken together, these studies imply that occlusion of the mouth region of the face should adversely affect the identification of emotions that significantly rely on the processing of the lower part of the face such as happiness (except for faces with Duchenne smiles), sadness and anger (Fischer et al., 2012; Kret & de Gelder, 2012), as well as surprise and disgust (Bassili, 1979). Bassili, (1979) suggested that the bottom of the face is utilized for the recognition of happiness, surprise, and disgust, whereas the opposite is true for sadness and fear. For identification of anger, both areas are equally important. Research has argued that emotion recognition from the face is actually rather complex. People process emotions in a featural manner, or a configural manner, or both. Whereas features refer to certain face parts, such as the eyes or mouth, configurations refer to relational information such as interrelationships between the mouth and the eyes. That is, certain emotions are not derived by looking at one region (Bombari et al., 2013; McKelvie, 1995; Prkachin, 2003). In a study where participants viewed intact, blurred or scrambled faces with different types of information (featural, configural, or both), happiness was found to be recognized more easily and rapidly than the other emotions irrespective of the conditions. This suggests that happiness is mainly recognized in a featural manner, by looking at the smiling mouth. Happiness recognition should therefore be significantly affected by covering the mouth unless there is a Duchenne smile which includes smiling of the eyes. Fear, too, was found to be recognized in a featural manner. Though fear is mainly identified by the big open eyes, the mouth is also instrumental in detecting fear (Bombari et al., 2013). Hence, when occluding the mouth, fear detection could be impaired, yet to a lesser degree compared to happiness. Sadness is recognized in a configural way (Bombari et al., 2013), eliciting longer fixations on the center of the face (Bombari et al., 2013) or the eye area (Bassili, 1979; Beaudry et al., 2014; Eisenbarth & Alpers, 2011; Wegrzyn et al., 2017). At the same time, eyebrows and mouth are used for the recognition of sadness (Calvo et al., 2014; Smith et al., 2005). Sadness recognition, then, might be affected by occlusion of the mouth, albeit to a lesser degree compared to happiness. Anger is identified both in a featural and configural way. The mouth is not necessarily a relevant feature for the recognition of anger (Bombari et al., 2013), as it involves contraction of the corrugator supercilii muscle, resulting in furrowing of the brow (Fischer et al., 2012). Anger should be less impaired by covering the mouth as the upper part of the face is focused when detecting anger. Yet, the mouth area was suggested to be the most useful for the recognition of surprise and both the nose and mouth area are used for the recognition of disgust (Calvo et al., 2014; Smith et al., 2005) so one could expect impairment of such emotions as a result of occlusion of the mouth.

Research on the effect of occlusion of particular face regions on emotion recognition accuracy is especially relevant in the context of understanding the effects of face masks under the ongoing COVID-19 pandemic. By covering about 60% of the face that is pertinent to emotional expression (Carbon, 2020; Freud et al., 2020), face masks interfere with the recognition of its wearer’s emotional state (Freud et al., 2020; Marini et al., 2021), resulting in misinterpretation of emotions (Carbon, 2020). Carbon showed that applying a mask on faces resulted in a significant accuracy decline in reading basic emotions such as happiness, anger, disgust and sadness except for fear and neutral. For confidence ratings, along with others, fear and neutral emotions also revealed significant decreases. Further analyses demonstrated some non-random patterns of confusions between the emotional states in unmasked faces, which became more pronounced for the masked faces. For example, disgusted faces were misinterpreted as angry in almost 40% of the cases (2% of the cases in the no mask condition). In addition, Carbon found that different emotional states were more frequently confused with a neutral state in the masked condition. A similar study, this time with medical students, showed that students made more errors when faces were presented with face masks for emotions of happiness, anger, and sadness, but not for fear (Bani et al., 2021). Yet, in another study (Ramachandra & Longacre, 2022), half of the participants were shown pictures of the whole face of a woman and the other half was shown only the eyes of the same woman expressing the same emotions (similar to a mask covering the mouth). The results revealed a significant difference in emotion recognition between full face versus eyes conditions for all emotions except sadness and distress. While the recognition accuracy was the best for happiness in the face condition, it was the best for surprise in the eyes condition, and it was the worst for distress in both face and eyes conditions. Surprise and sadness were better in the eyes-only condition. Other studies demonstrated that face masks impact recognition of expressions significantly involving lower face features the most (disgust, anger), and emotions involving upper face features the least (fear, surprise) (McCrackin et al., 2021). In sum, previous evidence on the role of face masks in emotion recognition suggests that, overall, masks impede emotion recognition; however, this effect for specific emotions varies from study to study. Fear seems to be the least affected emotion and all other emotions seem to be adversely influenced by occlusion of the mouth, yet there seems to be no consensus as to how other emotions are affected. One reason for inconsistencies between studies could be the use of different stimuli in experiments across papers. Carbon (2020), for instance, showed that recognizing emotions of elderly faces was much harder than that for middle-aged or young faces. Hence, age differences among the models used in these studies might be one factor. Also, using different models with different ability to express emotions may play a role. For example, Carbon used a professional data set with models, who can clearly pose emotional states, and found relatively high accuracy in recognition of emotions in masked faces. Moreover, sample characteristics might have contributed to the observed inconsistencies in the literature. An examination of eye movements showed that while Western individuals’ eye fixations were scattered evenly across the faces, Eastern individuals mainly focused on the eyes (Jack et al., 2009). In addition, there is research that demonstrates that as individuals’ exposure to masks increases, they actually get better at reading emotions (Barrick et al., 2020). Hence, the timing of these studies may matter. Those which were closer to the beginning of the pandemic may demonstrate lower accuracy ratings compared to more recent ones.

Overall, previous research indicates that emotional states are harder to decipher and easily confused when a target occludes their mouth region by wearing a face mask. It must be noted, however, that the abovementioned studies focused on mask versus no mask conditions generally applying standard often white medical masks. However, in reality, individuals use different types of face coverage with different appearance (e.g., patterned and colored masks). There are only a few studies that investigated the effect of non-medical and non-white masks on emotion recognition. Employing bi-state electrochromic displays, Genç et al. (2020) created smart masks—a *Mouthy Mask* (reproducing the image of the mask wearer's mouth) and a *Smiley Mask* (using an emoji instead of a representation of a mouth). Results showed that individuals on average preferred visualizations representing the wearer's mouth as a means to mitigate facial expression occlusion (Genç et al., 2020). Though specific emotions were not studied in this research, overall perceived understandability of the emotions significantly improved when smart face masks were used. Further, Marini et al. (2021) demonstrated that in contrast to standard medical face masks, transparent masks significantly increase the capability to recognize emotional expressions (Marini et al., 2021) for all emotions (happiness, sadness, fear) except for neutral. In fact, no significant differences were observed in accuracy between non-mask and transparent mask conditions across all emotions.

### Emotion recognition: effects of mask patterns and color

In the current study, we aimed to examine factors related to mask appearance that might play a role in recognition of emotional states. We contend that the pattern (angular vs. curvy patterns) or the color (black vs. white) of the mask may serve as an additional input when reading emotions. To our knowledge, no researcher to date has studied the effect of masks with different patterns or color and how they affect emotion recognition.

Our motivation to study the effects of angularity versus curvature is based on research in multiple disciplines, from art and esthetics to neurobiology, from visual cognition to social psychology and marketing. These works highlighted differences in perception of angular versus curved shapes (Carbon, 2010; Hussain, 1972; Palumbo et al., 2015). The curved over sharp preference was claimed to be a basic visual primitive evolutionary-based function (Carbon, 2010). A cross-cultural study demonstrated that curvature is one of the dimensions that drives preference of people from different cultures around the world (Gómez-Puerto et al., 2018). Angular stimuli were found to be associated with threat (Aronoff et al., 1988, 1992) and aggressive emotions (Hevner, 1935; Lundholm, 1921; Poffenberger & Barrows, 1924). Furthermore, Bar and Neta (2006) suggested that curvature preference could be due to threat avoidance. Palumbo et al. (2015) found that angular polygons were associated with negative emotions, whereas curved polygons were associated with positive emotions. Blazhenkova and Kumar (2018) also found associations between abstract angular shapes with negative emotions and curved shapes with positive emotions. Different attributions for angularity versus curvature in terms of emotional valence and arousal (e.g., unpleasant, agitating, and harsh vs. pleasant, gentle, and quiet) were also reported in earlier research on esthetic perception (Lundholm, 1921; Poffenberger & Barrows, 1924). Besides, angularity versus curvature convey different meanings and benefits in marketing literature. For instance, while angular brand logos denote conflict and aggressiveness, round logos are seen as harmonious and gentle (Jiang et al., 2015; Zhang et al., 2006). As angularity depicts hardness and aggressiveness, a juice coming in an angular package (compared to a curvy one) leads consumers to experience the product taste as more intense (Becker et al., 2011). Blazhenkova and Dogerlioglu-Demir (2020) investigated the effects of pills’ shape (angular vs. curvy) on the perceived efficacy of the medicine, bodily sensations and emotions and found that the angular pills trigger more activations in the body compared to curvy pills. While angularity was found to be linked with an energizing effect, roundness was associated with a calming effect. Further, angularity was linked with negative emotions such as anxiety, fear, anger, and irritation. However, a positive relief emotion was evoked by curved shapes (Blazhenkova & Dogerlioglu-Demir, 2020). The literature also suggested that curved shapes resemble facial emotions expressing happiness (curves in cheeks, smiling mouth) whereas angular shapes resemble angry expressions (v-shaped angles in the eyebrows) (Aronoff et al., 1992; Bassili, 1979). Infants’ rounded head and facial features were associated with warmth and protectiveness (Papanek, 1995), while the sharp or angular shapes such as sharp edges of the teeth of a tiger are associated with danger. These results therefore suggest that the effect of angularity versus curvature is a robust finding and it may be related to face perception. Thus, we expected to find that masks with angular patterns may enhance the perception of anger (and other negative emotions), whereas masks with curved patterns may enhance the perception of happiness (and other positive emotions).

Another characteristic of visual appearance related to emotional processing is color, and we also expected to see its effect on recognition of emotions on masked faces. Past research tested associations between colors and emotions and found that whereas white is seen as a happy color (Clarke & Costall, 2008), black is perceived as a sad color (Cimbalo et al., 1978). Similarly, white is associated with positivity and joy and black is associated with negativity, depression, evil, and even death (Sliburyte & Skeryte, 2014). White is generally linked to sincerity as it is associated with characteristics such as cleanness, simplicity, and peace. Black also stands for sophistication, glamor and power (Labrecque & Milne, 2012). In the fashion world, black is the most worn color for dressy occasions (Funk & Nelson, 2006). Black expresses status and elegance (e.g., black tie events, little black dresses and tuxedos) (Labrecque & Milne, 2012). Although these associations have been observed across cultures (for instance, black is consistently associated with ‘expensive’ and ‘powerful’ across cultures, De Bortoli & Maroto, 2001), culture specific linkages have also been demonstrated (Hanada, 2018). Additional meaning associations of “formal” (Brazil, Colombia, PRC, and Taiwan) and “masculine” (Austria, Hong Kong, the United States) were evident in some countries (Madden et al., 2000). In all countries, white is associated with “peaceful,” “gentle,” and “calming.” Some cultures associate “beautiful” (Brazil, Hong Kong, PRC, United States) with white (Madden et al., 2000). White is associated with cleanliness and purity as well as emptiness (Saito, 1996) and emotionlessness (Hanada, 2018) in Asian cultures and mourning particularly in China (De Bortoli & Maroto, 2001). Based on this literature, in our study, we anticipated to observe that black masks (and darker patterned masks) enhance the perception of negative emotions whereas white masks (and lighter patterned masks) may enhance the perception of positive emotions in masked faces.

Furthermore, we expected that individual difference variables (such as emotional processing traits and attitudes toward masks) may play a role in emotion recognition in masked faces. Previous research has indicated that attitudes toward masks may influence emotion recognition. In a recent study by Biermann et al. (2021), a stronger negative bias was found in happiness and trustworthiness judgments of faces with masks for those who attribute less protective effect to masks, have a lower experienced risk concerning COVID-19, and those who see face mask wearing as a burden. Further, for trustworthiness appraisals, the negative bias was stronger for those who tend to comply less with face mask wearing rules (Biermann et al., 2021). Further, face masks by themselves may also have negative associations with danger (e.g., pollution, disease) and covering the face even by a non-mask fabric (e.g., scarf) may trigger the feelings of insecurity (i.e., someone is hiding true emotions or having bad and unclear intentions). There is also evidence showing that certain face coverings (e.g., niqab) are associated with an out-group, and impact the readability of the emotions of women wearing them (Fischer et al., 2012). Though some emotional signals may still be perceived, observers may be biased toward seeing more negative emotions, when they are guessing about what is going on beneath the veil. No studies to date, however, examined the effect of mask appearance on the attitudes toward masks. Attitudes toward these different masks (patterned or different colors) may relate to emotion recognition, and we aimed to explore this.

Finally, we examined individual differences in emotional processing (i.e., richness of emotional experiences or anxiety) in relation to the perception of emotions on masked faces. The previous literature suggested that individual differences may predict emotion recognition in general, and also in faces when only eyes are visible. For example, persons with higher overall social competence were found to be better at identifying unmasked expressions, while persons with lower trait extraversion and higher trait agreeableness were better at recognizing masked expressions (McCrackin et al., 2021). Besides, people with difficulties in emotional processing such as those with high functioning autism or Asperger syndrome were found to have impaired emotion recognition from the eyes alone more than other populations (Baron-Cohen et al., 1997b). More recent studies showed that higher degree of autistic traits predicted a greater difficulty in recognizing emotions both with and without masks (Pazhoohi et al., 2021; Ramachandra & Longacre, 2022). Anxiety was also found to be related to altered emotion recognition, so that individuals with high trait anxiety were shown to identify fear significantly better. However, other emotions did not differ across low trait anxiety and high trait anxiety groups (Surcinelli et al, 2006). Other reports showed that anxious individuals tend to have a negative bias and to identify neutral faces as angry (Deighton & Traue, 2007; Demenescu et al., 2010). No studies examined anxiety in relation to emotion recognition of masked faces. As the mere presence of masks may trigger anxiety (Saint & Moscovitch, 2021) due to the ambiguity the mask creates (people hiding face and possibly intentions), we expected even more negative bias in anxious individuals reading emotions from masked faces.

Our current research aimed to investigate the effect of individual difference variables on emotion recognition and attitudes toward masks when masks of different appearance are used. Consistent with previous studies, we expected to find that people with richer emotional experiences would have better recognition of emotions on masked faces, and people with emotional difficulties such as anxiety may have impaired emotion recognition in masked faces. Moreover, we aimed to further explore whether individual differences in emotional processing relate to emotion recognition depending on mask appearance.

At first blush, because previous studies indicate that recognition of basic emotions is better for whole faces than it is for partial faces, it may be predicted that wearing any face mask might interfere with accurate emotion evaluations. We further suggest that the patterns on the mask as well as its color may serve as an additional factor when judging emotions. The present research aims to fill this knowledge gap by investigating recognition of emotions as well as potential confusion of emotional states due to wearing patterned or colored face masks. To our knowledge, our research constitutes the first step toward understanding how the visual appearance of occluders may interfere with emotional processing of faces.

### Hypothesis 1 (mask effects on recognition performance)

(H1a) The presence of masks impairs emotion recognition. Consistent with the previous literature, we expect that emotional states on masked faces are less recognizable, resulting in lower recognition accuracy, longer response times, and lower confidence in recognition. (H1b) Different types of mask (curvy, angular, plain white, or plain black) differently affect emotion recognition when compared to unmasked faces. Pattern and color on masks may influence the accuracy, confidence and speed of emotion recognition. The previous literature does not provide enough evidence on which to base specific predictions about masks with different designs, but we expect to see some differences among them.

### Hypothesis 2^1^ (design effects on emotional valence)

Angular and black designs result in more negative emotion perceptions whereas curvy and white designs would yield more positive emotion perceptions. That is, we expect to observe more accurate recognition of negative emotions such as Anger for angular and black mask designs, and more accurate recognition of positive emotions such as Happiness for curved and white mask designs. Besides, we expect that emotions for faces in masks with black colors and angular patterns are more likely to be misattributed as negative, whereas emotions for faces in masks with white colors and curved patterns are more likely to be misattributed as positive. This prediction is based on the above-reviewed literature on the perception of angularity versus curvature and its relation to emotional processing.

### Hypothesis 3 (individual differences)

Individual differences in emotional processing and attitudes toward masks are related to recognition of emotions in faces with masks with different designs. Emotion recognition in people with higher anxiety and negative attitudes toward mask wearing is more impaired by masks. We do not have specific predictions about the relationship between individual differences and recognition of emotional states in faces with specific patterns on masks but we expect possible differences.

## Method

### Participants

Participants were students from a Turkish university studying in the English language. They were recruited via the university’s Sona system and received bonus course credits for their participation. Initially, 157 students participated in the study. To assure that the motivation and engagement of the participants were sufficient, we used several exclusion criteria. Among the respondents, 10 failed the attention check question (‘Now please ignore this question and mark 'agree'’; and a further seven did not disagree with another attention check statement ‘I am not a human’ and therefore were excluded from the analysis. In addition, we excluded 5 participants whose very long response times suggested they were not really paying attention to the study (their average page submit time exceeded 2*SD*^2^ higher than the grand mean). The 137 participants (91 females, 18–29 years old, mean age 22 years old) were retained for the analysis. The study was conducted in May 2021, more than a year after the beginning of the pandemic, and the university was using fully remote education at the time (the campus was closed). Please also note that in Turkey, where we conducted our study, COVID-19 policies have been moderately stringent. For instance, certain workplaces, schools, and recreational areas were closed, yet travel was allowed with proof of accommodation reservations (Dogerlioglu-Demir et al, 2021; Hale, et al., 2021). Even in the beginning of the pandemic (January 2020), the rate of use of masks was high though there was no legal obligation to do so (60% of those surveyed wore masks when in public) (Akdeniz et al, 2020). Later, in May 2020, it was made mandatory to use face masks in some cities. Then, in September 2020, it was made mandatory to use them in all cities in public. This legal obligation still continues (Topal & Arslan Topal, 2021).

### Procedure

Participants completed the *Masks’ rating task* as well as a few individual differences self-report measures assessing attitudes toward masks and emotional processing. They provided informed consent and completed the study online via Qualtrics software.

### Materials

#### Masks’ rating task

**Fig. 1:**
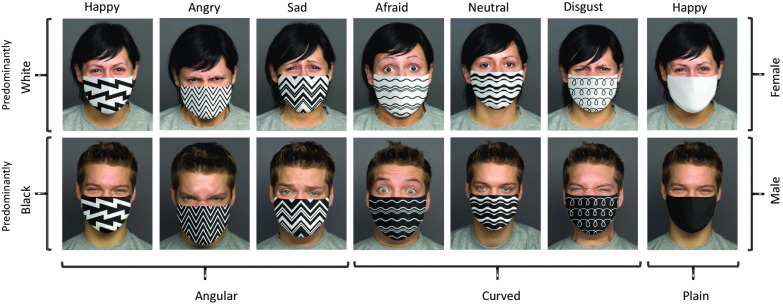
Examples of stimuli used in the masks’ rating task

Face images were taken from FACES, a database comprising faces of women and men expressing different emotions (Ebner et al., 2010), the same as used by Carbon (2020). FACES is a validated database created for scientific research, using trained models clearly posing the emotions. It was especially important to have clearly recognizable faces in the unmasked condition as a baseline to see the specific impairments in emotion recognition due to masks. Therefore, we only included 1 young male and 1 young female face. Masks on faces were edited with Photoshop (Fig. 1) using pictures with angular and curved patterns obtained from iStockphoto. Masks’ rating task consisted of 168 *Masked Faces* trials: (Sex—2 [male, female], Emotions—6 [afraid, angry, disgust, happy, neutral, sad], Patterns—7 [3 angular, 3 curved, plain], Color—2 [predominantly white, predominantly black—we included purely black and white masks, and for the patterned masks we applied inversion]), 14 *Masks Only* trials (Patterns—7, Color—2), and 12 *Unmasked Faces* trials (Sex—2, Emotions—6). *Masked Faces* trials were administered first, followed by *Masks Only* trials, and finally by *Unmasked Faces* trials.

Following Carbon’s (2020) procedure, each participant was exposed to the complete set of stimuli one after another. Though each participant completed all the blocks of trials in the same order (masked faces, masks only, and unmasked faces), the order of trials was randomized within each block. This order of the blocks was required for our study because the ‘masks only’ block could not go before the ‘masked faces’ block, as we did not want to prime our participants with mask designs during the emotion recognition task. Moreover, to prevent the influence of unmasked faces on the recognition of emotions on masked faces, the unmasked faces had to be displayed as the last block. For each trial, a stimulus and the respective questions were presented on one separate page. The response time was not restricted but recorded, and participants proceeded to the next trial by clicking the ‘next’ button. Specifically, we measured ‘page submit’ time, which counts the time in seconds rounded to the nearest millisecond (Qualtrics, 2020) from the onset of the page until the “next” button is clicked. This provides a rough estimate of the overall time spent for each trial. Besides, the overall time spent on each page was used as an estimate of potential survey respondent fatigue. Each trial was presented on a single page but included two tasks. Therefore, we additionally used the ‘first click’ time metric which counts how many seconds were spent before the respondent first clicked the page. This provides a rough estimate of time spent for the first task—selecting the emotion among the options. The timing of the questions was hidden from the participants.

In each of the *Masked Faces* or *Unmasked Faces* trials, participants viewed a masked face and had to indicate a person’s emotional state (choose from a list of 7 emotions: Afraid, Angry, Disgust, Happy, Neutral, Sad, and Surprise). Next, they had to indicate their confidence in assessing emotion from 1 (very unconfident) to 7 (very confident). Both accuracy and response time (time to first click, time to page submit) were recorded. Note that we did not include an “I don’t know” answer in the emotion recognition task. Further, participants were forced to respond in each trial by picking one of the options in the given list of emotions. This was by design, as we wanted to specifically see the patterns of mistakes people make when exposed to different mask designs.

In *Masks Only* trials, participants were asked to ‘indicate the extent the pattern on the above mask is…’ (1) on the scale from 1 (Extremely Curvy) to 7 (Extremely Angular), (2) on the scale from 1 (Extremely Black) to 7 (Extremely White). These questions were included to check the shape and color manipulations. Next, participants rated their preferences: ‘Imagine that you have to attend a social event indoors, and there is a rule to wear a mask. How likely is it that you would wear a mask with this pattern?’ on the scale from 1 (Not likely at all) to 7 (Very much likely).

#### Range and differentiation of emotional experience scale (RDEES)

This self-report scale assesses individual differences in emotional complexity defined as having emotional experiences that are broad in range and well differentiated (Kang & Shaver, 2004). On a 5-point Likert scale, participants rated their agreement with 14 statements from the 2 subscales: *Emotional Differentiation* (e.g., ‘I am aware of the different tones or subtleties of my various emotions’; *α* = 0.79) and *Emotional Range* (e.g., ‘I experience a wide range of emotions’; *α* = 0.82).

#### Short State-Trait Anxiety Inventory (SSTAI)

The abbreviated version of Spielberger State-Trait Anxiety Inventory (STAI; Spielberger et al., 1970) assesses individual anxiety levels and includes 5-items per *State* (STAIS-5; *a* = 0.91) and *Trait Anxiety* (STAIT-5; *α* = *0.8*6) scales (Zsido et al., 2020).

#### Face Mask Perceptions Scale (FMPS)

The FMPS (Howard, 2020) is a 32-item questionnaire that assesses negative attitudes toward mask wearing on eight dimensions: *Comfort* (e.g., ‘Face masks disrupt my breathing’’; *α* = 0.91; note, all Cronbach's alpha values for FMPS scales here and below are reported based on Howard, 2020), *Efficacy Doubts* (e.g., ‘Face masks provide few health benefits’; *α* = 0.86), *Access* (e.g., ‘Face masks are too expensive’; *α* = 0.86), *Compensation* (e.g., ‘I already social distance’; *α* = 0.86), *Inconvenience* (e.g., ‘It is hard to develop the habit of wearing a face mask’; *α* = 0.83), *Appearance* (e.g., ‘Face masks look silly’; *α* = 0.97), *Attention* (e.g., ‘Face masks make people seem untrustworthy’; *α* = 0.94), *Independence* (e.g., ‘I do not like feeling forced to do something’; *α* = 0.89). In addition, we developed 5 new questions for a new *Emotion* scale, assessing difficulty in reading emotions (e.g., ‘I can't understand people's emotions clearly when they wear face masks’; ‘I can't express my emotions clearly when I wear face masks’; ‘I do not feel emotionally connected with people who wear face masks’; ‘I think people cannot clearly understand my emotions when I wear face masks’; ‘People cannot feel emotionally connected with me when I wear face masks’; *α* = 0.92 based on the data of the current study). Besides, we included an unvalidated 4-items scale, *Face Mask Perceptions,* FMP +*,* (e.g., ‘People should wear face masks in public’; *α* = 0.63 based on the data of the current study), assessing positive attitudes toward masks not only in the context of COVID but also in general (Howard, 2020).

#### Mask wearing habits and COVID-19 risks estimation

Participants responded to ‘Within the 6 months, how often have you worn a face mask *outdoors* when going into public?’ and ‘Within the 6 months, how often have you worn a face mask *indoors* when going into public?’ on the scale from 1 (Never) to 7 (Every time). They also responded to ‘Please indicate the current situation with COVID-19 in the country where you reside now. How would you estimate the risk with COVID-19 in your country?’ on the scale from 1 (Very risky) to 7 (Not risky at all).

## Results

### Recognition performance

#### Analytic strategy

Each of the four primary dependent variables (recognition accuracy, confidence, page submit time, and time to first click) was subjected to two analyses.

Firstly, to assess the overall effect of different types of mask on general emotion recognition (H1a and H1b), we analyzed performance on each trial (i.e., trials were treated as cases) with a generalized linear mixed model, with Emotion (Happy, Angry, Sad, Afraid, Neutral, Disgust), Mask Type (Plain White, Plain Black, Angular, Curvy, and No Mask), and their interaction as fixed factors; participants were allowed a random effect on the intercept. Emotion was deviation coded, with Neutral faces as the untested category; Mask Type was dummy coded with No Mask as the reference category. With this analysis, the effect of interest is the main effect of Mask Type: the parameter estimates for this effect test whether performance for that type of mask was different from performance for the No Mask condition, averaged across all Emotions.

Secondly, to examine whether different mask designs vary in the extent to which they impair recognition performance (H1a and H2), we ran another model again analyzing performance on each trial: this time, for each trial, that participant’s mean performance for that emotion in the No Mask condition was entered as a covariate. This is conceptually equivalent to analyzing each mask’s effect on performance relative to the No Mask condition, i.e., how much that type of mask impaired performance. Emotion, Mask Color (White or Black), and Mask Pattern (Plain, Angular, or Curvy), and all their interactions were entered as fixed factors, and participants were allowed a random effect on the intercept as before. In these analyses, we were interested in any effects involving Mask Color or Mask Pattern, since these would indicate that different masks impaired performance (relative to No Mask) to different extents. For example, a significant Mask Color × Emotion interaction would indicate that Black versus White masks have different effects on performance for some emotions.

Analyses were conducted in R 4.0.2 (R Core Team, 2020) using the *lme4* package (1.1–23, Bates et al., 2015), with the help of *emmeans* (1.4.8, Lenth, 2020) and *car* (3.0–8, Fox & Weisberg, 2019). The Laplace approximation was used, and Wald tests were used for hypothesis testing.

#### Recognition accuracy

**Fig. 2:**
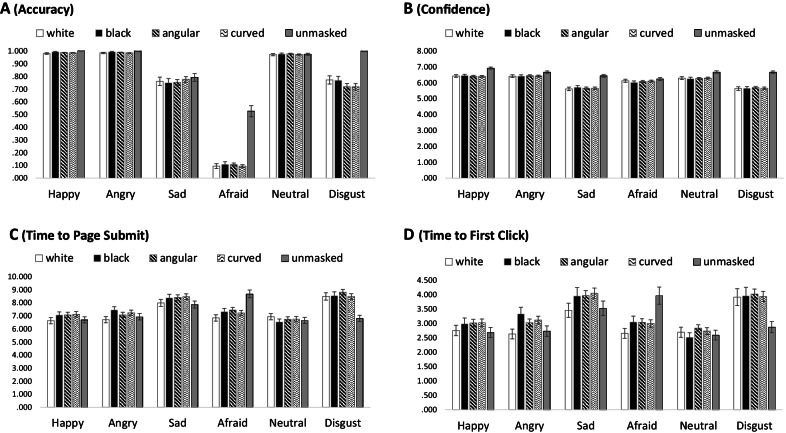
Recognition accuracy, confidence, and response time for masks’ rating task. *Note*: Error bars represent standard errors of the mean

We first assessed the overall effect of different types of mask on general emotion recognition accuracy, as described above. Recognition accuracy was subjected to a generalized linear mixed model with a binomial distribution and logit link. We excluded 37 trials with high Pearson residuals. The main effect of Mask Type—the effect of interest for this analysis—was significant, *χ*^2^ (4) = 139.71, *p* < 0.001, so that faces wearing White (*M* = 0.879, coefficient = − 3.942, *p* < 0.001), Black (*M* = 0.913, coefficient = − 3.575, *p* < 0.001), Angular (*M* = 0.894, coefficient = − 3.796, *p* < 0.001), and Curvy masks (*M* = 0.881, coefficient = − 3.927, *p* < 0.001) were all recognized less accurately than faces with no mask (*M* = 0.997). There was also a main effect of Emotion, *χ*^2^ (5) = 5025.81, *p* < 0.001, such that Happy faces were recognized more accurately than the mean, whereas Afraid and Sad faces were recognized less accurately than the mean. There was also an interaction, *χ*^2^ (20) = 178.83, *p* < 0.001; simple effects analysis with *emmeans* revealed a significant simple main effect of Mask Type for Afraid (*F* (4) = 60.258, *p* < 0.001), Happy (*F*(4) = 8.301, *p* < 0.001), and Disgust faces (*F*(4) = 9.569, *p* < 0.001) and a marginal effect for Angry faces (*F*(4) = 2.117, *p* = 0.076); the simple main effect of Mask Type was not significant for Neutral (*F*(4) = 0.482, *p* = 0.749) or Sad faces (*F*(4) = 0.916, *p* = 0.453). Together Fig. 2A, this suggests that masks impaired recognition accuracy, but only for Happy, Angry, Afraid, and Disgust expressions.

We next assessed the differences among mask types’ effects on recognition accuracy. No main effects or interactions involving Mask Pattern or Mask Color approached significance (all *p*s > 0.18), indicating that all mask types had equivalent effects on recognition accuracy, for all emotions.

To account for possible speed/accuracy trade-off (we are grateful to an anonymous reviewer for raising this possibility), we re-ran the above model with page submit time included as a covariate: The results were unchanged.

#### Emotion recognition confidence

As for recognition accuracy above, we first examined whether masks reduced participants’ confidence in their overall emotion judgements, relative to their confidence in their judgements about No Mask faces. We estimated the model with a Poisson distribution and log link (confidence was reflected for analysis; the means and coefficients given below are on the original, unreflected response scale). We excluded 113 trials with high Pearson residuals.

There was a main effect of mask type, *χ*^2^ (4) = 206.445, *p* < 0.001. Fixed effect coefficients showed that White (*M* = 6.12, coefficient = − 0.311, *p* < 0.001), Black (*M* = 6.11, coefficient = − 0.314, *p* < 0.001), Angular (*M* = 6.12, coefficient = − 0.307, *p* < 0.001), and Curvy masks (*M* = 6.12, coefficient = − 0.308, *p* < 0.001) all reduced confidence relative to the No Mask condition (*M* = 6.62). There was also a main effect of Emotion, *χ*^2^ (5) = 1317.100, *p* < 0.001, showing that Afraid and Sad faces yielded lower confidence than the mean, and Happy faces yielded higher confidence than the mean. There was also an interaction, *χ*^2^ (20) = 65.482, *p* < 0.001; the simple main effect of Mask Type was significant for all Emotions (*F*s > 2.46, *p*s < 0.044) except Afraid (*F*(4) = 1.137, *p* = 0.337. Together with Fig. 2B, this suggests that masks impair confidence, relative to No Mask, for all the other emotions.

We then examined whether different types of mask had different effects on confidence, relative to the No Mask condition. No main effects or interactions involving Mask Color or Pattern approached significance, *p*s > 0.528, suggesting that all mask designed had similar effects on confidence, for all Emotions.

#### Page submit time

Since these data were collected online, there was considerable variation in page submit times: participants participating at home would presumably have been occasionally interrupted, and some probably took the study more seriously than others. In order to yield more acceptable model fit, trials with page submit times greater than 2.5 standard deviations above or below the mean for that condition were excluded from this set of analyses. This does not greatly affect the conclusions.

We first examined whether masks in general affected page submit times, compared to the No Mask condition. We ran a generalized linear mixed model with an inverse Gaussian distribution and log link. The analysis then proceeded as for recognition accuracy above. The important main effect of Mask Type was significant, *χ*^2^ (4) = 52.517, *p* < 0.001, such that participants responded more slowly to faces wearing Black (*M* = 7.60 s, coefficient = 0.231, *p* = 0.015), Angular (*M* = 7.58 s, coefficient = 0.214, *p* = 0.002), and Curvy masks (*M* = 7.59 s, coefficient = 0.221, *p* = 0.002) than they did to No Mask faces (*M* = 7.37 s); but not to faces wearing White masks (*M* = 7.31 s, coefficient = − 0.063, *p* = 0.485). There was also a main effect of Emotion, *χ*^2^ (5) = 1207.555, *p* < 0.001, such that participants responded to Afraid and Sad faces slower than the mean, and responded to Angry, Happy and Disgust faces faster than the mean. There was also a significant interaction, *χ*^2^ (20) = 194.128, *p* < 0.001; the simple main effect of Mask Type was significant for all emotions (*F*s > 3.77, *p*s < 0.016), but not for Neutral faces (*F*(4) = 0.225, *p* = 0.925). Together with Fig. 2C, this suggests that masks affected page submit time, but only for emotional expressions: They did not affect page submit times for Neutral expressions.

We next tested the differences among mask types. There was a significant main effect of Mask Pattern, *χ*^2^ (2) = 9.778, *p* = 0.008, such that participants responded slightly faster to faces in Plain masks (*M* = 6.76 s) than they did to faces in Angular (*M* = 6.94 s, Tukey-corrected *p* = 0.012) or Curvy masks (*M* = 6.93 s, *p* = 0.020). There was also a Mask Pattern × Color interaction, *χ*^2^ (2) = 8.915, *p* = 0.012, such that participants responded somewhat faster to faces in plain white masks (*M* = 6.59 s) than they did to faces in Plain Black masks (*M* = 6.92 s), *F* (1) = 9.558, *p* = 0.002, whereas there were no differences between White and Black Angular, *F* (1) = 0.870, *p* = 0.351, or Curvy masks, *F* (1) = 0.242, *p* = 0.622. No other effects involving Mask Pattern or Mask Color approached significance, *p*s > 0.144.

#### Time to first click

Times to first click were trimmed of outlier trials as for page submit times above and analyzed using a model with an inverse Gaussian distribution and a log link.

The main effect of Mask Type was significant, *χ*^2^ (4) = 26.785, *p* < 0.001, such that participants responded more slowly to faces wearing Black (*M* = 3.26 s, coefficient = 0.073, *p* = 0.035), Angular (*M* = 3.28 s, coefficient = 0.080, *p* = 0.002), and Curvy masks (*M* = 3.27 s, coefficient = 0.078, *p* = 0.003) than they did to No Mask faces (*M* = 3.03 s); but not to faces wearing White masks (*M* = 2.98 s, coefficient = − 0.015, *p* = 0.665). There was also a main effect of Emotion, *χ*^2^ (5) = 420.395, *p* < 0.001, such that participants responded more quickly than the mean to Angry and Happy faces, and more slowly than the mean to Afraid and Sad faces. There was a significant interaction, *χ*^2^ (20) = 57.246, *p* < 0.001; the simple main effect of Mask Type was significant for Angry (*F*(4) = 3.397, *p* = 0.009), Afraid (*F*(4) = 5.625, *p* < 0.001), and Disgust faces (*F*(4) = 7.137, *p* < 0.001), marginal for Sad faces (*F*(4) = 2.088, *p* = 0.080), and nonsignificant for Happy (*F*(4) = 1.506, *p* = 0.198) and Neutral faces (*F*(4) = 1.318, *p* = 0.261). Together with Fig. 2D, this suggests that participants generally responded more quickly to masked than No Mask faces when the faces were Afraid, and more slowly to masked faces when the faces were Angry or Sad.

Again, we tested for differences among mask types. We again saw the Mask Pattern × Color interaction, *χ*^2^ (2) = 8.658, *p* = 0.013, such that participants responded faster to faces in Plain White masks (*M* = 2.89 s) than they did to faces in Plain Black masks (*M* = 3.19 s), *F* (1) = 8.778, *p* = 0.003, whereas there were no differences between White and Black Angular, *F* (1) = 0.120, *p* = 0.729, or White and Black Curvy masks, *F* (1) = 0.740, *p* = 0.390. No other effects involving Mask Pattern or Mask Color approached significance, *p*s > 0.478.

#### Confusion between different emotional states

Similar to Carbon (2020), we examined the specific confusions of different emotional states in unmasked and all masked conditions. To compare the frequencies of selecting each emotion for each of the six posed expressions, we used descriptive analysis and reported the percentages from cross-tabulation tables (see confusion matrix in Table 1). Overall, most of the emotional states posed by *unmasked faces* were not misinterpreted. One exception was Sad that was mostly confused with Afraid (18.61%) and less so with Disgust (8.03%); this is somewhat similar to Carbons’ findings (Sad was mainly confused with Disgust, 20.3%). The second exception was Afraid that was misinterpreted as Surprise in almost half of the cases (45.99%). Afraid-Surprised confusion is a common finding in the literature (Bassili, 1979). Note, we added the Surprise answer option while we did not actually have a Surprise-posing face. We did not find Angry misinterpretation as Disgusted, reported by Carbon, or any other considerable confusions for the unmasked faces.

**Table 1:**
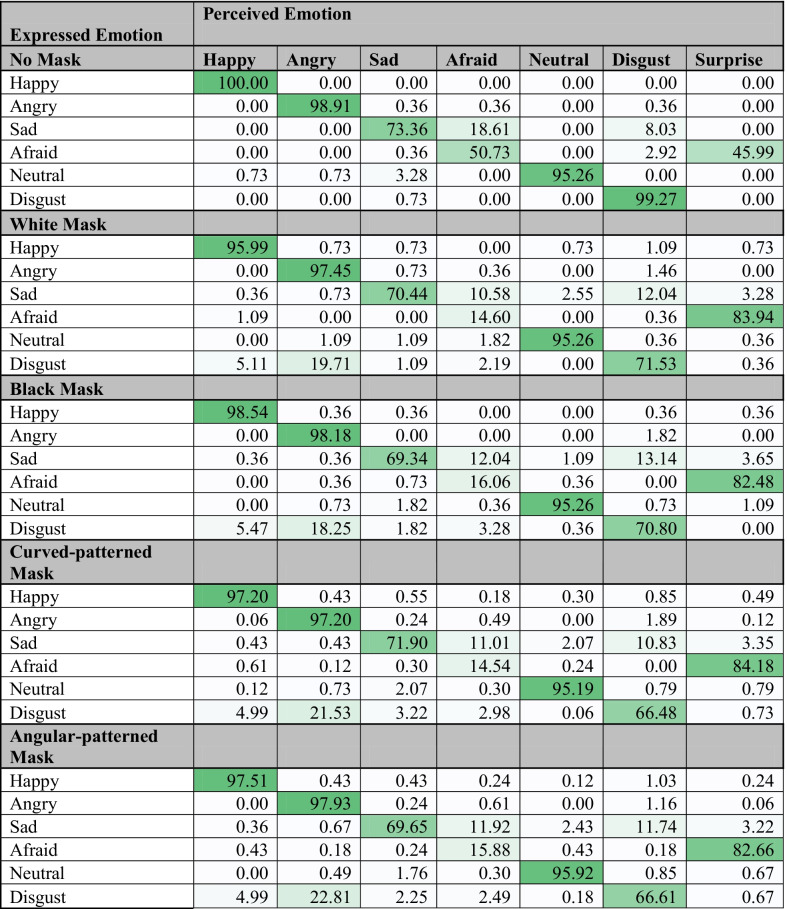
Confusion matrix of emotions

The darkness of the green indicates the degree of correspondence between the expressed and perceived emotion

For all kinds of *masked faces*, we observed very similar patterns of misinterpretations. Similar to Carbon, we found that for masked faces, the characteristic confusions became more outstanding. Sad, overall, was more frequently confused with both Disgust and Afraid, but at a comparable rate (10–13% each). Afraid became drastically confused with Surprise (82–84%). Similar to Carbon, we found that Disgust became confused with Angry (12–22%), though it was less pronounced than in Carbon’s study (37.8%). Unlike Carbon, we did not find that masked faces were more frequently misinterpreted as Neutral in the masked condition. Also, inconsistently with Carbon, Happy and Angry emotional states were rarely confused with others in either masked or unmasked conditions.

Given the very high accuracy of emotion recognition even in the masked conditions, we suspected that our participants were learning due to multiple exposure to the same masked faces. To examine this possibility, we conducted a nominal logistic regression with *Participant* as categorical random variable, *Order* as a continuous predictor, and their interaction; emotion recognition accuracy *Score* was the categorical response. The results demonstrated a strong and significant Participant effect (log-likelihood *χ*^2^ [136] = 1391.84, *p* < 0.001) indicating variability in participants’ responses. The effect of Order was moderate and also significant (*χ*^2^ [1] = 5.43, *p* = 0.02), indicating that, overall, participants learned in the process, however not a lot. The interaction was strong and significant (*χ*^2^ [136] = 216.24, *p* < 0.001), indicating that participants learned differently: while some improved, some did not change or even worsened their performance.

### Pattern, color and preference ratings of masks without faces

One-sample t tests on the ratings of *Angularity* demonstrated all angular patterns were rated as significantly higher in angularity than chance (test value = 4), *ps* < 0.001. All round patterns were rated as significantly lower in angularity than chance, *ps* < 0.001. Black mask angularity ratings did not differ from chance; however, White mask was rated as significantly lower in angularity (*p* = 0.039), similar to round patterned masks.

The similar analysis of *Color* ratings revealed that in all pairs with normal and color-inverted patterns, one was rated as significantly whiter and the other as less white than chance (test value = 4), all *ps* < 0.001 except for one pattern (Shown in Fig. 1—Angular, Predominantly White and Sad), consistently judged as predominantly white, but only marginally significantly (*p* = 0.082). Black mask was rated as significantly less white than chance (*p* < 0.001), and White mask was rated as significantly more white than chance (*p* < 0.001). White mask was perceived as significantly predominantly white than black mask (*p* = 0.001), whereas Angular and Curved patterns did not significantly differ in terms of the perceived color.

Repeated measures ANOVA revealed a significant effect of mask appearance on a *preference to mask wearing*, *F* (563.212, 3.902) = 144.327, *p* < 0.001 (*η*_*p*_^2^ = 0.515). Preference to wear a Black mask (*M* = 6.10, *SD* = 1.526) was the highest compared to other masks (all *ps* < 0.001). Preference to wear a White mask (*M* = 5.38, *SD* = 1.997) was higher than for masks with Angular (*M* = 3.09, *SD* = 1.85) and Curved (*M* = 2.87, *SD* = 1.78) patterns (both *ps* < 0.001). Preference to wear a mask with Angular patterns only tended to be higher than for Curved (*p* = 0.085).

A Friedman test indicated that mask appearance significantly affected *preference to wear each type of mask*, *χ*^2^ (3) = 194.473, *p* < 0.001. Bonferroni-corrected Wilcoxon tests showed that preference to wear a Black mask (*Med* = 7.00) was the highest compared to other masks (all *ps* < 0.001). Preference to wear a White mask (*Med* = 6.00) was higher than for masks with Angular (*Med* = 2.83) and Curved (*Med* = 2.50) patterns (both *p*s < 0.001). Preference to wear a mask with Angular patterns only tended to be higher than for Curved (*p* = 0.079).

### Emotion recognition and individual differences self-reports

We used Pearson’s correlation analysis to examine the relationships between emotion recognition and individual differences measures (Table 2).

**Table 2:** Correlations between emotion recognition and individual differences self-reports

	FMPS	FMPS1	FMPS2	FMPS3	FMPS4	FMPS5	FMPS6	FMPS7	FMPS8	FMPS9
ANG ACC	* − .143*	− .179^*^	− .107	− .122	.045	− .119	− .123	* − .154*	− .097	* − .156*
CUR ACC	− .171^*^	− .205^*^	− .133	− .13	.08	− .116	* − .167*	− .173^*^	* − .161*	− .170^*^
WHITE ACC	* − .161*	− .201^*^	− .123	− .200^*^	.015	− .079	− .115	− .134	* − .159*	− .229^**^
BLACK ACC	− .113	− .134	* − .156*	− .016	.003	− .073	− .101	− .077	− .1	− .068
NOMASK ACC	− .024	− .016	− .062	− .099	.037	.027	.01	− .028	− .064	− .028
ANG CONF	− .174^*^	* − .142*	− .113	− .191^*^	− .101	− .14	* − .159*	− .183^*^	− .065	* − .165*
CUR CONF	− .177^*^	− .139	− .134	− .192^*^	− .098	− .136	* − .163*	− .191^*^	− .061	− .175^*^
WHITE CONF	− .189^*^	− .129	* − .149*	− .219^*^	− .081	* − .147*	− .188^*^	− .212^*^	− .075	− .184^*^
BLACK CONF	− .181^*^	* − .166*	− .094	− .178^*^	* − .148*	− .134	* − .164*	− .178^*^	− .061	* − .16*
NOMASK CONF	.035	.014	.031	− .065	.03	.076	.068	− .058	.065	− .02
ANG FC	.1	.019	.077	.105	.081	.077	.026	.171^*^	.086	.088
CUR FC	.244^**^	*.149*	.174^*^	.188^*^	.085	.197^*^	.185^*^	.310^**^	.205^*^	.266^**^
WHITE FC	.131	.07	.098	*.167*	.049	.092	*.157*	.168^*^	.036	.132
BLACK FC	.069	.075	.08	.112	.081	.038	.019	.06	.002	.14
NOMASK FC	− .076	− .073	− .066	− .002	− .007	− .072	− .067	− .051	− .09	− .02
ANG PS	.226^**^	.112	.236^**^	*.166*	.085	.226^**^	.118	.288^**^	.180^*^	.206^*^
CUR PS	.293^**^	.180^*^	.256^**^	.212^*^	.06	.257^**^	.206^*^	.390^**^	.233^**^	.295^**^
WHITE PS	*.166*	.079	*.167*	.212^*^	.056	.13	*.15*	.215^*^	.069	.141
BLACK PS	*.146*	.124	.185^*^	*.149*	.09	.14	.062	.134	.058	.197^*^
NOMASK PS	.017	.011	.05	.023	− .079	.032	− .006	.079	.003	.023
WHITE PREF	− .059	− .079	− .111	0.01	− .034	− .076	− .105	− .027	.072	− .119
BLACK PREF	− .183^*^	− .123	− .153	− .169^*^	− .002	− .291^**^	− .226^**^	− .119	− .043	− .169^*^
ANG PREF	.185^*^	.02	.134	.114	.011	.196^*^	.177^*^	.264^**^	.204^*^	*.148*
CUR PREF	.234^**^	.096	.175^*^	.13	.014	.218^*^	.221^**^	.295^**^	.249^**^	*.158*

	FMP +	Outdoor	Indoor	Risks	STAIS	STAIT	EMO-R	EMO-D
ANG ACC	− .047	.02	.104	− .02	* − .149*	− .172^*^	− .043	− .026
CUR ACC	− .034	.013	.086	.028	− .126	− .170^*^	− .08	− .046
WHITE ACC	− .044	− .01	.037	.064	* − .164*	− .226^**^	− .137	− .076
BLACK ACC	.047	.052	.03	− .01	− .034	− .133	− .049	.074
NOMASK ACC	.034	.107	.102	.014	* − .147*	− .011	− .045	− .08
ANG CONF	.106	.005	.194^*^	.083	− .062	− .11	.091	.128
CUR CONF	.112	− .005	.204^*^	.071	− .071	− .131	.082	.11
WHITE CONF	.118	− .045	.217^*^	.111	− .066	− .128	.069	.112
BLACK CONF	.112	.02	*.163*	.028	− .056	− .082	.104	.133
NOMASK CONF	.11	.058	.153	.1	− .021	− .018	*.162*	.043
ANG FC	− .127	.077	− .084	− .005	− .002	.051	.079	− .014
CUR FC	− .112	0.1	− .029	− .049	.071	.032	.025	− .051
WHITE FC	* − .151*	.103	− .084	− .075	− .059	− .066	− .117	− .087
BLACK FC	− .04	.049	− .181^*^	.004	− .14	− .083	− .022	− .169^*^
NOMASK FC	− .017	− .055	− .116	.105	− .191^*^	− .114	* − .155*	− .001
ANG PS	* − .153*	.108	− .128	− .063	.056	.084	.11	− .015
CUR PS	− .113	.114	− .034	− .065	.141	.074	.033	− .03
WHITE PS	− .189^*^	.098	− .123	* − .143*	.007	− .051	− .071	− .084
BLACK PS	− .113	.089	− .178^*^	− .059	− .095	− .08	.021	− .169^*^
NOMASK PS	.001	.035	− .077	.035	− .09	− .048	− .003	.017
WHITE PREF	.039	− .025	.028	− .009	.036	− .073	− .026	.046
BLACK PREF	.201*	.068	.108	− .086	.043	.13	.131	.06
ANG PREF	− .023	− .006	− .271^**^	.169^*^	.096	.054	.008	− .046
CUR PREF	− .003	− .029	− .255^**^	*.156*	.200^*^	.086	.026	.029

**Correlation is significant at the 0.01 level (2-tailed), shown in bold. *Correlation is significant at the 0.05 level (2-tailed), shown in bold. ^+^Correlation is significant at the 0.1 level (2-tailed), shown in italics. FMPS scales: 1—Comfort, 2—Efficacy doubt, 3—Access, 4—Compensation, 5—Inconvenience, 6—Appearance, 7—Attention, 8—Independence, 9—Emotion (new). ANG, Angular; CUR, Curved; ACC, Accuracy; CONF, Confidence; FC and PS, time to first click and page submit; PREF, Preference

Notably, scores distribution analysis (FMPS: *M* = 2.77, *SD* = 1.14; MPS + : *M* = 22.57, *SD* = 1.13; Risks Estimate: *M* = 2.56, *SD* = 1.67; Mask Wearing Outdoors: *M* = 6.55, *SD* = 1.05; Mask Wearing Indoors: *M* = 5.92, *SD* = 1.73) indicates that, overall, our Turkish student sample had rather positive attitudes toward masks and high wearing habits. In fact, another study conducted with 3040 Turkish university students in April 2020 reported use of masks among university students was 50%, much higher than expected as there was no legal obligation to do so in the beginning of the pandemic.

#### Attitudes toward masks

The findings indicate that, overall, *negative attitudes toward masks* (higher scores on FMPS) tended to be negatively related to accuracy in emotion recognition only for masked but not unmasked faces. At the same time, FMPS positively correlated with the time of emotion recognition in faces with curved and angular masks. Consistent trends were observed for separate FMPS scales (see Table 2). FMPS negatively correlated with frequency of wearing masks indoors (*r* = − 0.214, *p* = 0.013). Wearing masks indoors positively correlated with Confidence in emotion recognition.

Interestingly, *preferences* to wear Black masks (that were the most prevalent as shown in the previous analysis) negatively correlated with FMPS, but preferences to wear the least preferred (patterned) masks positively correlated with FMPS. At the same time, preferences to wear patterned masks were negatively related to actual wearing masks indoors and perceived risks of COVID. FMP + (assessing positive attitudes toward wearing masks) positively correlated with preference for Black mask wearing. Preferences to wear White masks were positively correlated with higher accuracy (*r*s ranged between 0.247 and 0.313; *p*s ≤ 0.004) and confidence (*r*s ranged between 0.256 and 0.293; *p*s ≤ 0.003) in recognition of all kinds of masked but not unmasked faces. Those who preferred Black masks also had higher confidence (*r*s ranged between 0.200 and 0.210; *p*s ≤ 0.019) in recognition of all kinds of masked but not unmasked faces.

Based on these observations, we estimated a path model to test the hypothesis that higher FMPS scores, lower frequency of wearing masks indoors, and lower estimated risk of COVID were associated with poorer accuracy and lower confidence in recognizing masked but not unmasked faces, and with preferences to wear patterned but not plain masks. Our logic was that individuals with a less positive attitude toward masks and mask wearing might be more distracted by masks when judging others’ expressions (the reverse causation is also quite plausible in this case), and might tend to think of masks more as fashion accessories than necessities, and so prefer more decorative rather than clinically styled masks. The model was run in JASP’s (JASP Team, 2021) implementation of *lavaan* (Rosseel, 2012); standard errors were estimated using 10,000 bootstrap samples. FSMP score, frequency of indoor mask wearing, and estimated COVID risk were predictors and allowed to covary. Mean recognition accuracy for all mask types and recognition accuracy for unmasked faces were covarying outcomes, as were mean confidence for all masked faces and for unmasked faces, and mean preference to wear all patterned masks and to wear all plain (white or black) masks. Based on modification indices, we also allowed confidence for masked faces and preference to wear plain faces to covary. Due to the large number of modelled paths, model fit was good: *χ*^2^ (11) = 19.80, *p* = 0.048; RMSEA = 0.076; SRMR = 0.065; CFI = 0.959; GFI = 0.999.

FMPS score showed a marginal negative relationship with recognition accuracy for masked faces, standardized coefficient *β* = − 0.150, *p* = 0.081, but no relationship with accuracy for unmasked faces, *β* = − 0.000, *p* = 0.999; similarly, FMPS showed a marginal negative relationship with confidence for masked faces, *β* = − 0.149, *p* = 0.078, but no relationship with confidence for unmasked faces, *β* = 0.074, *p* = 0.385; FMPS significantly predicted preference to wear pattered masks *β* = 0.174, *p* = 0.034, but not plain masks, *β* = − 0.128, *p* = 0.139. Frequency of indoor mask wearing predicted confidence for both masked, *β* = 0.181, *p* = 0.033, and unmasked faces, *β* = 0.184, *p* = 0.033; it negatively predicted preference for wearing patterned, *β* = − 0.229, *p* = 0.006, but not for plain masks, *β* = 0.046, *p* = 0.597. Estimated risk of COVID-19 positively predicted preference for patterned, *β* = − 0.163, *p* = 0.041, but not plain masks, *β* = − 0.052, *p* = 0.539.

Of course, this model is exploratory and post hoc, and our sample size is small for a model of this complexity. But, these results provide some provisional support for our hypothesis that individuals with more negative attitudes toward masks and mask wearing are likely to prefer more decorative masks and may have more difficulties understanding the expressions of people wearing masks.

#### Individual differences in emotions

Consistent with our expectations, higher anxiety, especially trait anxiety, was associated with lower accuracy in recognizing emotions on masked faces (*r*s ranged between − 0.170 and − 0.226; *p*s ≤ 0.047, except Black). State and trait anxiety were positively associated with several scales of FMPS and at the same time with FMP + (*r* = 0.237, *p* = 0.005). Emotional Range correlated with both state (*r* = 0.210, *p* = 0.014) and trait (*r* = 0.261, *p* = 0.002) anxiety, and also with FMPS comfort scale (*r* = 0.206, *p* = 0.016).

### Sex differences

Participant’s sex, and all its possible interactions, were added as fixed factors in the generalized mixed models described above. Male participants were slightly faster to submit pages, *χ*^2^ (1) = 4.859, *p* = 0.028; otherwise, no effect involving sex approached significance (all *p*s < 425). There were no sex differences in mask preferences (both sexes preferred black masks).

Mann–Whitney tests of ranks revealed significant sex differences, favoring females, in Emotional Differentiation [*U* = 1272.000, *p* < 0.001; males: *M* = 3.68, *SD* = 0.58, females: *M* = 4.09, *SD* = 0.63)] and in Emotional Range [*U* = 1449.500, *p* = 0.003; males: *M* = 3.64, *SD* = 0.61, females: *M* = 3.98, *SD* = 0.71)], and no other significant differences in other self-report measures.

## General discussion

Our study explored the effects of face masks’ designs on emotion recognition. We examined emotion recognition in faces uncovered and covered by masks of different patterns (curved vs. angular) and colors (white and black). Consistent with the literature (Carbon, 2020; Freud et al., 2020; Marini et al., 2021) and our Hypothesis 1a, we found that the presence of masks impaired emotion recognition. Overall, accuracy and confidence dropped, and reaction time increased, for masked faces compared to unmasked faces. Contrary to our expectations (Hypothesis 1b), we did not find clear effects of mask pattern or color on emotion recognition. Faces covered by masks with different patterns and amount of white or black colors were recognized approximately at the same rate. The appearance of the mask, in terms of pattern and color, does not seem to greatly affect the extent to which it impairs emotion recognition. Yet, while all mask types had equivalent effects on recognition accuracy, participants responded somewhat faster to faces in plain white masks. Though the response time data should be interpreted very cautiously, our data indicate that white masks may have less of a disruptive effect. Possibly, patterned and black masks may impede recognition more than white masks at least in terms of speed, since more salient objects capture more attention and therefore may distract the observer. Future research may address this question by systematically testing the efficiency of face processing with different complexity of patterns and different colors of masks, as well as for faces with different ethnicities or complexions.

Inconsistent with Hypothesis 2, we did not find that angular and black designs consistently lead to better recognition of negative emotions and more negative misattributions, or that curvy and white designs yielded better recognition of positive emotions and more positive misattributions. The emotions were confused similarly in all masked faces compared to unmasked ones; we did not observe any negative or positive biases depending on the mask design. Overall, these results indicate that mask design did not significantly contribute to specific misinterpretations of emotions in masked faces. Our findings also suggest that people may be quite successful in ignoring irrelevant visual information while focusing on reading emotions from the eyes only.

It may be the case that mask design does not matter for the emotion recognition of masked faces, but it is also possible that we did not detect the effect for a number of reasons. Note that we utilized rather complex patterns on masks (e.g., zigzags and wavy). Though our participants perceived these shapes as intended (e.g., zigzag as angular and wavy as curvy), simpler and more obvious designs (e.g., one big circle, one triangle) could have made the perception of angular versus curvy more salient. Subsequent studies should investigate the effects of masks with simpler designs. In addition, the size and the density of the pattern may matter.

Notably, the difference between masked and unmasked conditions was not very large. Unlike previous research, we found a surprisingly high emotion recognition rate (ceiling effect; especially for Happy, Angry, and Neutral emotions). One reason could be using a scientific database with models clearly posing the emotions. Carbon (2020), who used the same database, also reported very high performance in recognition of unmasked faces (though he did not find significant differences between masked and unmasked face recognition for fearful and neutral emotional states). However, Carbon did not find such a high rate in recognition of masked faces. Notably, unlike Carbon, we used only the faces of young models. Carbon found that performance in recognizing emotions of elderly faces was lower than for middle-aged or young faces. Research suggested that young adults are better at recognizing emotions compared to older people (Grundmann et al., 2021). Since our participants were university students, the fact that the age of the models matched the age of the participants could also have made the task easier. In addition, our participants were students from a Turkish university. Previous studies showed that there may be differences across cultures in the way individuals read emotions. Some cultures (e.g., Eastern) were found to focus more on the eyes when reading emotions (Jack et al., 2009). Perhaps, the style of reading expressions might have facilitated emotion recognition in masked faces. Future research, therefore, should explore the proposed relationships by employing other samples (various cultures as well as a wider range of ages) and a broader range of stimuli (age-wise as well as trained vs. untrained models). Another reason for high performance can be repeated exposure to the same faces that could have increased the recognition of emotions. Notably, participants did not see unmasked faces before completing masked faces’ emotion recognition task, so high performance cannot be simply due to the recognition of previously seen unmasked faces. Yet, we found a small learning effect: an increase in emotion recognition accuracy depending on the order of a trial. Our results indicate that people may improve the accuracy of masked faces' emotion identification just from the repeated exposure within a short-term timeframe (our task took less than 20 min). Similarly, previous studies showed that individual differences in mask exposure influenced the use of visual cues from the face. A longitudinal study (two controlled experiments 6 months apart) assessed the perceived emotional similarity between a pair of unmasked faces (Barrick et al., 2020). As mask exposure increases, there is an increase in the eye cue use. This literature indicates that face perception is malleable and longer exposure to masked faces may actually change the way we read emotions. The longer people interact with others that wear masks, the more they learn to focus on visual cues from the eye area of the face, whereas there is some evidence that during pandemics, people may have learned to recognize masked faces better with time, which may partially explain increasing performance in recognition.

Remarkably, we observed some discrepancy between confidence and accuracy ratings. On the one hand, confidence ratings showed more clear differences between masked versus unmasked faces’ than recognition performance, i.e., the Mask Type effect on confidence was larger than the Mask Type effect on accuracy. On the other hand, there were less differences in confidence ratings between the emotions. The confidence in recognition of different emotional states was relatively comparable, indicating that confidence ratings may reflect a somewhat general estimate, by the participant, of their ability to understand emotions and be less sensitive to the differences between the emotions.

Finally, we expected to find the relationship between individual difference measures and emotion recognition (Hypothesis 3). As predicted, *attitudes* toward masks were related to performance in reading emotions. People with negative attitudes toward masks appeared to be less accurate and less confident in their recognition of emotions on masked faces. We cannot tell, though, whether negative attitudes lead to worse performance or vice versa. Notably, this relationship did not exist for unmasked faces, so negative attitudes toward masks were not related to emotion recognition skills in general. People frequently wearing masks indoors were more confident in emotion recognition, but again, only for masked faces. Thus, the attitudes and mask wearing behavior were specifically related to sensitivity to emotions on masked but not unmasked faces.

Our research suggests that certain individual traits and attitudes may predict the ability to recognize emotions on masked but not unmasked faces. It is possible that the selective impairment in processing masked emotions may be related to certain individual differences. As some literature suggests, reading emotions from isolated face regions may be specifically impaired in some populations (e.g., autistic: Baron-Cohen et al., 1997b; Pazhoohi et al., 2021; Ramachandra & Longacre, 2022). Possibly, occluding mouth regions may be disadvantageous for individuals with specific traits. Future studies should explore how people with superior ability for recognizing faces (super recognizers; Noyes, et al., 2017; Russell et al., 2009) are affected by masks. Consistent with our expectations, higher anxiety was associated with lower emotion recognition accuracy in masked faces as well as with negative attitudes toward masks. At the same time, anxiety was positively associated with FMP + (positive attitudes toward masks). Thus, both positive and negative attitudes may be potentially motivated by anxiety. Overall, our findings demonstrate the existence of the relationship between individual differences in emotional processing, attitudes toward masks, and reading emotions from masked faces, though it may be rather complex. Future studies need to further search for the individual differences that predict emotional processing in masked faces.

We observed some consistent relationships among *preferences* for specific mask appearances, attitudes toward masks, and emotion recognition. The majority of participants demonstrated positive attitudes toward mask wearing and preferred non-patterned black and white masks. Those who preferred white unpatterned masks had higher accuracy and confidence in recognition of all kinds of masked but not unmasked faces. Similarly, those who preferred black (also unpatterned) masks had higher confidence in recognition of all kinds of masked but not unmasked faces. Interestingly, those with negative attitudes toward masks preferred patterned masks. So, it may be that they perceive masks as entertaining, funny or a fashion accessory rather than a protective medical item. At the same time, people frequently wearing masks indoors (which is more important than wearing masks outdoors in the context of COVID-19) did not prefer angular and curved masks, which may indicate that they did not perceive patterned masks seriously. Interestingly, those with negative attitudes toward masks also tended to be slower in making decisions about emotional states especially when the patterned masks were worn. Possibly, because they perceived patterned masks more as a clothing item rather than as a medical device (medical masks are usually non-patterned masks) therefore these participants took more time to aesthetically examine them.

Our study has several limitations. We must acknowledge that ‘Time to page submit’ and ‘Time to first click’ are not very precise measures of response time for the emotional identification task; however, they provide rough estimate of time spent on the task (Qualtrics, 2022). Notably, the data from these time measures were generally consistent with the accuracy data. The response time data were of secondary importance for our analyses, yet we believe they meaningfully contribute to the understanding of emotion recognition processes for masked faces. Despite the fact Qualtrics and other web-based reaction time surveys have attracted considerable skepticism regarding the accuracy of their time measurement, recent research suggests that online reaction time data are more trustworthy than was previously believed (Armitage & Eerola, 2020; Semmelmann & Weigelt, 2017).

We also acknowledge the limitations of having only two faces as stimuli, which reduces the generalizability of our conclusions. Furthermore, using the same faces and masks and repeating them over the trials creates the possibility of carryover effects. Even if only masked faces were repeated, participants still could focus on responding coherently across trials. However, our examination of learning effects shows only a small increase in accuracy; besides, it was quite high from the very beginning. Notably, our manipulation included not only different emotional expressions but also different types of masks, so in order to show carryover effects, participants would have had to monitor several Emotional states and Mask patterns. Even if they completely ignored differences in mask patterns, the strategy of memorizing the answers for each emotional state and responding coherently across 168 randomized trials seems unlikely, as it creates an exceptionally high working memory demand. Visual working memory has a maximum capacity of about four objects and tends to store integrated objects rather than individual features (Luck & Vogel, 1997). Moreover, the time interval separating stimuli and additional distractors (evaluation of confidence), as well as the similarity between the stimuli and other emotions and masks (as reported by previous research, working memory capacity reductions correlate with increases in sample-test similarity; Awh et al., 2007), minimizes the possibility of carryover effects. Even though visual working memory can be influenced by the previous trial (i.e., proactive interference), performance mainly depends on the contents of the current trial (Makovski & Jiang, 2008). When the participants were presented with the same unmasked faces, the accuracy significantly increased; therefore, even if the manipulation was clear for them, they did not try to be consistent with the previous responses on the same faces and changed their answers. Besides, we used only two faces as stimuli since the main goal of the study was the comparison of emotion recognition on faces covered by different types of masks (also in comparison to the baseline unmasked face). Therefore, we wanted to minimize the variation between the faces, but only manipulate the presence and types of masks. As actors’ ages were closer to our subject pool's age, age similarity could enhance emotion recognition due to an “in-group” advantage (Elfenbein & Ambady, 2002). Also, reading the emotional status of younger faces was found to be easier (Carbon, 2020). Thus, using only young faces provided us with a more conservative test of mask effects.

Further, the curved and angular masks included in our research may not fully capture the differences between angular versus curved designs. Possibly, the lack of differences between recognition of emotions on angular versus curved masks is due to selection of these specific mask appearances. Our study calls for future research using different faces (including faces of different age and cultural groups) and different types of mask designs.

The COVID-19 pandemic has resulted in multiple paradigm shifts in society (Howe et al., 2020), creating habits such as wearing masks that are likely to stay even after the pandemic subsides (Sheth, 2020). Such behavior patterns are likely to have long-term effects on how people perceive socialization and engage in social interactions. In the near future, we may observe changes in attitudes toward masks, such as a perception of masks as a routine of daily life and a fashion statement. Thus, we expect to see further diversification of mask designs. More studies, therefore, are needed to examine how both mask appearance and attitudes toward masks may affect emotion recognition as well as how they may interact.

## Data Availability

The datasets generated and/or analyzed during the current study will be available in the OSF repository, upon the acceptance of the manuscript.

## Footnotes

1 We do not present hypotheses regarding the main effect of emotion, nor the variations of mask effects between the emotions. Our review showed that the literature is quite inconsistent, and the effect of concealing part of the face varies considerably depending on the stimuli used. In our study, we used only 2 faces; therefore, any observed differences between different emotions are unlikely to be informative or generalizable.

2 The outliers were removed since the response time data was used in the analysis.

## References

[CR1] Akdeniz G, Kavakci M, Gozugok M, Yalcinkaya S, Kucukay A, Sahutogullari B (2020). A survey of attitudes, anxiety status, and protective behaviors of the university students during the COVID-19 outbreak in Turkey. Frontiers in Psychiatry.

[CR2] Argyle M (1970). Eye-contact and distance: A reply to Stephenson and Rutter. British Journal of Psychology.

[CR3] Armitage J, Eerola T (2020). Reaction time data in music cognition: Comparison of pilot data from lab, crowdsourced, and convenience Web samples. Frontiers in Psychology.

[CR4] Aronoff J, Barclay AM, Stevenson LA (1988). The recognition of threatening facial stimuli. Journal of Personality and Social Psychology.

[CR5] Aronoff J, Woike BA, Hyman LM (1992). Which are the stimuli in facial displays of anger and happiness? Configurational bases of emotion recognition. Journal of Personality and Social Psychology.

[CR6] Awh E, Barton B, Vogel EK (2007). Visual working memory represents a fixed number of items regardless of complexity. Psychological Science.

[CR7] Bani M, Russo S, Ardenghi S, Rampoldi G, Wickline V, Nowicki S, Strepparava MG (2021). Behind the mask: Emotion recognition in healthcare students. Medical Science Educator.

[CR8] Bar M, Neta M (2006). Humans prefer curved visual objects. Psychological Science.

[CR9] Baron-Cohen S, Jolliffe T, Mortimore C, Robertson M (1997). Another advanced test of theory of mind: Evidence from very high functioning adults with autism or Asperger syndrome. Journal of Child Psychology and Psychiatry.

[CR10] Baron-Cohen S, Wheelwright S, Jolliffe AT (1997). Is there a “language of the eyes”? Evidence from normal adults, and adults with autism or Asperger syndrome. Visual Cognition.

[CR11] Barrick, E., Thornton, M. A., & Tamir, D. (2020). *Mask exposure during COVID-19 changes emotional face processing*. https://psyarxiv.com/yjfg3/download?format=pdf 10.1371/journal.pone.0258470 PMC8509869 34637454

[CR12] Bassili JN (1979). Emotion recognition: The role of facial movement and the relative importance of upper and lower areas of the face. Journal of Personality and Social Psychology.

[CR13] Bates D, Maechler M, Bolker B, Walker S (2015). Fitting linear mixed-effects models using lme4. Journal of Statistical Software.

[CR14] Beaudry O, Roy-Charland A, Perron M, Cormier I, Tapp R (2014). Featural processing in recognition of emotional facial expressions. Cognition & Emotion.

[CR15] Becker L, van Rompay TJL, Schifferstein HNJ, Galetzka M (2011). Tough package, strong taste: The influence of packaging design on taste impressions and product evaluations. Food Quality and Preference.

[CR16] Biermann M, Schulze A, Unterseher F, Atanasova K, Watermann P, Krause-Utz A, Stahlberg D, Bohus M, Lis S (2021). Trustworthiness appraisals of faces wearing a surgical mask during the Covid-19 pandemic in Germany: An experimental study. PLoS ONE.

[CR17] Blais C, Fiset D, Roy C, Saumure Régimbald C, Gosselin F (2017). Eye fixation patterns for categorizing static and dynamic facial expressions. Emotion.

[CR18] Blais C, Roy C, Fiset D, Arguin M, Gosselin F (2012). The eyes are not the window to basic emotions. Neuropsychologia.

[CR19] Blazhenkova O (2017). Boundary extension in face processing. I-Perception.

[CR20] Blazhenkova O, Dogerlioglu-Demir K (2020). The shape of the pill: Perceived effects, evoked bodily sensations and emotions. PLoS ONE.

[CR21] Blazhenkova O, Kumar MM (2018). Angular versus curved shapes: Correspondences and emotional processing. Perception.

[CR22] Bombari D, Schmid PC, Schmid Mast M, Birri S, Mast FW, Lobmaier JS (2013). Emotion recognition: The role of featural and configural face information. Quarterly Journal of Experimental Psychology.

[CR23] Bruce V, Young A (1986). Understanding face recognition. British Journal of Psychology.

[CR24] Calvo MG, Fernández-Martín A, Nummenmaa L (2014). Facial expression recognition in peripheral versus central vision: Role of the eyes and the mouth. Psychological Research Psychologische Forschung.

[CR25] Carbon C-C (2010). The cycle of preference: Long-term dynamics of aesthetic appreciation. Acta Psychologica.

[CR26] Carbon C-C (2020). Wearing face masks strongly confuses counterparts in reading emotions. Frontiers in Psychology.

[CR27] Cimbalo RS, Beck KL, Sendziak DS (1978). Emotionally toned pictures and color selection for children and college students. The Journal of Genetic Psychology.

[CR28] Clarke T, Costall A (2008). The emotional connotations of color: A qualitative investigation. Color Research and Application.

[CR29] Corpuz JCG (2021). Adapting to the culture of “new normal”: An emerging response to COVID-19. Journal of Public Health.

[CR30] De Bortoli, M., & Maroto, J. (2001). *Colours across cultures: Translating colours in interactive marketing communications*. globalpropaganda.fresa.net. http://www.globalpropaganda.fresa.net/articles/TranslatingColours.pdf

[CR31] Deighton RM, Traue HC (2007). Emotion recognition patterns in patients with panic disorder. Depression and Anxiety.

[CR32] Demenescu LR, Kortekaas R, den Boer JA, Aleman A (2010). Impaired attribution of emotion to facial expressions in anxiety and major depression. PLoS ONE.

[CR33] Dogerlioglu-Demir K, Akpinar E, Ceylan M (2021). Combating the Fear of COVID-19 through shared accommodations: Does perceived human presence create a sense of social connectedness?. Journal of Consumer Behaviour.

[CR34] Ebner NC, Riediger M, Lindenberger U (2010). FACES—A database of facial expressions in young, middle-aged, and older women and men: Development and validation. Behavior Research Methods.

[CR35] Eisenbarth H, Alpers GW (2011). Happy mouth and sad eyes: Scanning emotional facial expressions. Emotion.

[CR36] Ekman P, Cole JR (1972). Universal and cultural differences in facial expression of emotion. Nebraska symposium on motivation, 1971.

[CR37] Elfenbein HA, Ambady N (2002). On the universality and cultural specificity of emotion recognition: A meta-analysis. Psychological Bulletin.

[CR38] Fischer AH, Gillebaart M, Rotteveel M, Becker D, Vliek M (2012). Veiled emotions: The effect of covered faces on emotion perception and attitudes. Social Psychological and Personality Science.

[CR39] Fisher K, Towler J, Eimer M (2016). Effects of contrast inversion on face perception depend on gaze location: Evidence from the N170 component. Cognitive Neuroscience.

[CR40] Fox J, Weisberg S (2019). An R companion to applied regression.

[CR41] Freud E, Stajduhar A, Rosenbaum RS, Avidan G, Ganel T (2020). The COVID-19 pandemic masks the way people perceive faces. Scientific Reports.

[CR42] Funk D, Nelson ON (2006). Colour and product choice: A study of gender roles. Management Research News.

[CR43] Genç, Ç., Colley, A., Löchtefeld, M., & Häkkilä, J. (2020, September). Face mask design to mitigate facial expression occlusion. In *Proceedings of the 2020 international symposium on wearable computers*, 40–44.

[CR44] Gilad S, Meng M, Sinha P (2009). Role of ordinal contrast relationships in face encoding. Proceedings of the National Academy of Sciences of the United States of America.

[CR45] Goldstein AG, Mackenberg EJ (1966). Recognition of human faces from isolated facial features: A developmental study. Psychonomic Science.

[CR46] Gómez-Puerto G, Rosselló J, Corradi G, Acedo-Carmona C, Munar E, Nadal M (2018). Preference for curved contours across cultures. Psychology of Aesthetics, Creativity, and the Arts.

[CR47] Grundmann F, Epstude K, Scheibe S (2021). Face masks reduce emotion-recognition accuracy and perceived closeness. PLoS ONE.

[CR48] Hale T, Angrist N, Goldszmidt R, Kira B, Petherick A, Phillips T (2021). A global panel database of pandemic policies (Oxford COVID-19 Government Response Tracker). Nature Human Behaviour.

[CR49] Hanada M (2018). Correspondence analysis of color–emotion associations. Color Research and Application.

[CR50] Hevner K (1935). Experimental studies of the affective value of colors and lines. The Journal of Applied Psychology.

[CR51] Howard MC (2020). Understanding face mask use to prevent coronavirus and other illnesses: Development of a multidimensional face mask perceptions scale. British Journal of Health Psychology.

[CR52] Howe DC, Chauhan RS, Soderberg AT, Buckley MR (2020). Paradigm shifts caused by the COVID-19 pandemic. Organizational Dynamics.

[CR53] Hussain MZ (1972). Effect of shape of medication in treatment of anxiety states. The British Journal of Psychiatry: THe Journal of Mental Science.

[CR54] Itier RJ, Alain C, Sedore K, McIntosh AR (2007). Early face processing specificity: It’s in the eyes!. Journal of Cognitive Neuroscience.

[CR55] JASP Team. (2021). JASP (Version 0.15.0) [Computer software].

[CR56] Jack RE, Blais C, Scheepers C, Schyns PG, Caldara R (2009). Cultural confusions show that facial expressions are not universal. Current Biology: CB.

[CR57] James TW, Huh E, Kim S (2010). Temporal and spatial integration of face, object, and scene features in occipito-temporal cortex. Brain and Cognition.

[CR58] Janik SW, Wellens AR, Goldberg ML, Dell’Osso LF (1978). Eyes as the center of focus in the visual examination of human faces. Perceptual and Motor Skills.

[CR59] Jiang Y, Gorn GJ, Galli M, Chattopadhyay A (2015). Does your company have the right logo? How and why circular- and angular-logo shapes influence brand attribute judgments. The Journal of Consumer Research.

[CR60] Kang S-M, Shaver PR (2004). Individual differences in emotional complexity: Their psychological implications. Journal of Personality.

[CR61] Kotsia I, Buciu L, Pitas L (2008). An analysis of facial expression recognition under partial facial image occlusion. Image and Vision Computing.

[CR62] Kret ME, De Gelder B (2012). A review on sex differences in processing emotional signals. Neuropsychologia.

[CR63] Labrecque LI, Milne GR (2012). Exciting red and competent blue: The importance of color in marketing. Journal of the Academy of Marketing Science.

[CR64] Lenth, R. (2020). *emmeans: Estimated marginal means, aka least-squares means*. R package version 1.4.8. Retrieved from, https://CRAN.R-project.org/package=emmeans.

[CR65] Luck SJ, Vogel EK (1997). The capacity of visual working memory for features and conjunctions. Nature.

[CR66] Lundholm H (1921). The affective tone of lines: Experimental researches. Psychological Review.

[CR67] Madden TJ, Hewett K, Roth MS (2000). Managing images in different cultures: A cross-national study of color meanings and preferences. Journal of International Marketing.

[CR68] Makovski T, Jiang YV (2008). Proactive interference from items previously stored in visual working memory. Memory & Cognition.

[CR69] Marini M, Ansani A, Paglieri F, Caruana F, Viola M (2021). The impact of facemasks on emotion recognition, trust attribution and re-identification. Scientific Reports.

[CR70] McCrackin S, Capozzi F, Mendell E, Provencher S, Mayrand F, Ristic J (2021). Recognition of emotions is affected by face masks. Journal of Vision.

[CR71] McKelvie SJ (1995). Emotional expression in upside-down faces: Evidence for configurational and componential processing. British Journal of Social Psychology.

[CR72] Okazaki S, Yamanami H, Nakagawa F, Takuwa N, Kawabata KJD (2021). Eyes compensate smile when wearing mask.

[CR73] Noyes E, Phillips PJ, O’Toole AJ, Bindemann M, Megreya AM (2017). What is a super-recogniser?. Face processing: Systems, disorders and cultural differences.

[CR74] Palumbo L, Ruta N, Bertamini M (2015). Comparing angular and curved shapes in terms of implicit associations and approach/avoidance responses. PLoS ONE.

[CR75] Papanek, V. J. (1995). *Green imperative*. Thames and Hudson. https://agris.fao.org/agris-search/search.do?recordID=US201300300688.

[CR76] Pazhoohi F, Forby L, Kingstone A (2021). Facial masks affect emotion recognition in the general population and individuals with autistic traits. PLoS ONE.

[CR77] Pellicano E, Rhodes G, Peters M (2006). Are preschoolers sensitive to configural information in faces?. Developmental Science.

[CR78] Poffenberger AT, Barrows BE (1924). The feeling value of lines. The Journal of Applied Psychology.

[CR79] Prkachin GC (2003). The effects of orientation on detection and identification of facial expressions of emotion. British Journal of Psychology.

[CR80] Qualtrics. (2020).* Qualtrics development company*. Provo, UT. Retrieved from: https://www.qualtrics.com.

[CR81] Qualtrics. (2022). *Timing question*. Retrieved from, https://www.qualtrics.com/support/survey-platform/survey-module/editing-questions/question-types-guide/advanced/timing/.

[CR82] R Core Team. (2020). *R: A language and environment for statistical computing*. R Foundation for Statistical Computing. https://www.R-project.org/.

[CR83] Ramachandra V, Longacre H (2022). Unmasking the psychology of recognizing emotions of people wearing masks: The role of empathizing, systemizing, and autistic traits. Personality and Individual Differences.

[CR84] Roberson D, Kikutani M, Döge P, Whitaker L, Majid A (2012). Shades of emotion: What the addition of sunglasses or masks to faces reveals about the development of facial expression processing. Cognition.

[CR85] Rosseel Y (2012). lavaan: An R package for structural equation modeling. Journal of Statistical Software.

[CR86] Russell R, Duchaine B, Nakayama K (2009). Super-recognizers: People with extraordinary face recognition ability. Psychonomic Bulletin & Review.

[CR87] Saint SA, Moscovitch DA (2021). Effects of mask-wearing on social anxiety: An exploratory review. Anxiety, Stress, & Coping.

[CR88] Saito M (1996). Comparative studies on color preference in Japan and other Asian regions, with special emphasis on the preference for white. Color Research and Application.

[CR89] Semmelmann K, Weigelt S (2017). Online psychophysics: Reaction time effects in cognitive experiments. Behavior Research Methods.

[CR90] Sheldon KM, Goffredi R, Corcoran M (2021). The glow still shows: Effects of facial masking on perceptions of Duchenne versus social smiles. Perception.

[CR91] Sheth J (2020). Impact of Covid-19 on consumer behavior: Will the old habits return or die?. Journal of Business Research.

[CR92] Silchenko K, Visconti LM (2021). Facemask: From pandemic to marketplace iconicity. Consumption Markets & Culture.

[CR93] Sliburyte L, Skeryte I (2014). What we know about consumers’ color perception. Procedia, Social and Behavioral Sciences.

[CR94] Smith ML, Cottrell GW, Gosselin F, Schyns PG (2005). Transmitting and decoding facial expressions. Psychological Science.

[CR95] Spielberger CD, Gorsuch RL, Lushene R (1970). STAI manual Palo Alto.

[CR96] Surcinelli P, Codispoti M, Montebarocci O, Rossi N, Baldaro B (2006). Facial emotion recognition in trait anxiety. Journal of Anxiety Disorders.

[CR97] Taylor S, Asmundson GJG (2021). Negative attitudes about facemasks during the COVID-19 pandemic: The dual importance of perceived ineffectiveness and psychological reactance. PLoS ONE.

[CR98] Topal M, Arslan Topal EI (2021). Determination of the estimated amounts of discarded face masks due to COVID 19 in Turkey. Pollution.

[CR99] World Health Organization. (2020).* Advice on the use of masks in the context of COVID-19*, WHO/2019-nCov/IPC_Masks/2020.3.

[CR100] Wegrzyn M, Vogt M, Kireclioglu B, Schneider J, Kissler J (2017). Mapping the emotional face. How individual face parts contribute to successful emotion recognition. PLoS ONE.

[CR101] Willis J, Todorov A (2006). First impressions: Making up your mind after a 100-ms exposure to a face. Psychological Science.

[CR102] Zhang Y, Feick L, Price LJ (2006). The impact of self-construal on aesthetic preference for angular versus rounded shapes. Personality & Social Psychology Bulletin.

[CR103] Zsido AN, Teleki SA, Csokasi K, Rozsa S, Bandi SA (2020). Development of the short version of the Spielberger state—Trait anxiety inventory. Psychiatry Research.

